# Altered Microbiota Diversity and Bile Acid Signaling in Cirrhotic and Noncirrhotic NASH-HCC

**DOI:** 10.14309/ctg.0000000000000131

**Published:** 2020-03-04

**Authors:** Svenja Sydor, Jan Best, Insa Messerschmidt, Paul Manka, Ramiro Vilchez-Vargas, Susanne Brodesser, Christina Lucas, Annemarie Wegehaupt, Chiara Wenning, Sophia Aßmuth, Simon Hohenester, Alexander Link, Klaas Nico Faber, Han Moshage, Francisco Javier Cubero, Scott L. Friedman, Guido Gerken, Michael Trauner, Ali Canbay, Lars P. Bechmann

**Affiliations:** 1 Department of Gastroenterology, Hepatology, and Infectious Diseases, Otto von Guericke University Hospital Magdeburg, Magdeburg, Germany;; 2 Department of Gastroenterology and Hepatology, University Hospital Essen, Essen, Germany;; 3 CECAD Research Center, CECAD Lipidomics Facility, University of Cologne, Cologne, Germany;; 4 Department of Medicine II, University Hospital, LMU Munich, Munich, Germany;; 5 Department of Gastroenterology and Hepatology, University of Groningen, University Medical Center Groningen, Groningen, The Netherlands;; 6 Department of Laboratory Medicine, University of Groningen, University Medical Center Groningen, Groningen, The Netherlands;; 7 Department of Immunology, Ophthalmology and ENT, Complutense University School of Medicine, Madrid, Spain;; 8 12 de Octubre Health Research Institute (imas 12), Madrid, Spain;; 9 Division of Liver Diseases, Department of Medicine, Icahn School of Medicine at Mount Sinai, New York, New York, USA;; 10 Division of Gastroenterology and Hepatology, Department of Internal Medicine III, Medical University of Vienna, Vienna, Austria;; 11 Department of Medicine, Ruhr University Bochum, University Hospital Knappschaftskrankenhaus Bochum, Bochum, Germany.

## Abstract

The precipitous increase in nonalcoholic steatohepatitis (NASH) is accompanied by a dramatic increase in the incidence of NASH-related hepatocellular carcinoma (HCC). HCC in NASH has a higher propensity to arise without pre-existing cirrhosis compared with other chronic liver diseases.

**METHODS:**

To identify the potential links between liver and gut in NASH-related hepatocarcinogenesis, we compared the gut microbiota and mediators of bile acid (BA) signaling in the absence or presence of cirrhosis through the analysis of stool and serum samples from patients with NASH non-HCC and NASH-HCC and healthy volunteers.

**RESULTS:**

Serum levels of total and individual BA were higher in NASH compared with healthy controls. Furthermore, serum levels of the primary conjugated BAs glycine-conjugated cholic acid, taurine-conjugated cholic acid, glycine-conjugated chenodeoxycholic acid, and taurine-conjugated chenodeoxycholic acid were significantly increased in cirrhotic vs noncirrhotic patients, independent of the occurrence of HCC. By contrast, serum FGF19 levels were higher in patients with NASH-HCC and associated with tumor markers as well as an attenuation of BA synthesis. Specific alterations in the gut microbiome were found for several bacteria involved in the BA metabolism including Bacteroides and Lactobacilli. Specifically, the abundance of Lactobacilli was associated with progressive disease, serum BA levels, and liver injury in NASH and NASH-HCC.

**DISCUSSION:**

Here, we demonstrate a clear association of the altered gut microbiota and primary conjugated BA composition in cirrhotic and noncirrhotic patients with NASH-HCC. Microbiota-associated alterations in BA homeostasis and farnesoid X receptor signaling, via FGF19, might thus contribute to fibrogenesis, liver injury, and tumorigenesis in NASH-HCC.

## INTRODUCTION

Worldwide, hepatocellular carcinoma (HCC) is the sixth most common malignancy and the second leading cause of cancer-related deaths (1). In the Western world, the incidence of HCC is increasing, with underlying fatty liver disease as a major risk factor. Nonalcoholic fatty liver disease (NAFLD) may progress to nonalcoholic steatohepatitis (NASH), which is characterized by inflammation and hepatocellular ballooning (2,3). NASH-cirrhosis is recognized as risk factor for HCC (4). However, a significant proportion of patients with NASH develop HCC in the absence of cirrhosis, which (5,6) is ascribed in part to the pathophysiological changes related to lipotoxicity, bile acid (BA) signaling, and inflammation (7–9).

We and others have shown that serum BA concentrations are elevated in advanced NASH (10–12). BA accumulation induces parenchymal liver injury and contributes to the progression of NAFLD (13,14). The cascade of progression from simple steatosis via NASH to HCC is believed to be orchestrated by a complex interplay within the gut-liver axis, involving serum BA and the intestinal microbiome. Several studies have identified BA as important molecules modulating the gut microbiome in metabolic disorders (15–17).

Primary BA are synthesized in hepatocytes from cholesterol in a multistep process involving cholesterol-7-alpha-hydroxylase (CYP7A1) and secreted into the bile. Its expression is regulated by the BA-activated nuclear receptor farnesoid X receptor (FXR). FXR and its hepatic and intestinal target genes NR0B2 encoding the small heterodimer partner and fibroblast growth factor 19 (FGF19), transcriptionally regulate BA *de novo* synthesis, secretion, and reabsorption in the enterohepatic circulation (18). In the intestine, BA are modified by bacteria, reabsorbed from the ileum, and returned to the hepatocytes through the enterohepatic circulation. Microbiome alterations may promote the progression of NAFLD (19).

Here, we have explored whether microbial composition might contribute to NASH-associated hepatocarcinogenesis and whether this depends on the presence of cirrhosis. Moreover, we characterized the mediators of BA signaling and identified the associated alterations in the gut microbiome in patients with NASH with or without HCC.

## METHODS

### Sample collection

Patients were recruited at the University Hospital Essen (UHE) from May 2015 until November 2017. All subjects provided informed written consent, and the UHE Ethics Committee (Institutional Review Board) approved the study (reference: 14-6044-BO). The study protocol conformed to the ethical guidelines of the Declaration of Helsinki.

Subjects were divided into 5 groups (NASH-non-HCC, NASH-non-HCC-cirrhosis, NASH-HCC, NASH-HCC-cirrhosis, and healthy controls). NASH was diagnosed by ultrasonography and/or according to histological features, when available, in the presence of obesity and/or metabolic syndrome.

HCC was diagnosed according to the EASL guidelines (20). Cirrhosis was diagnosed by histology or clinical signs of portal hypertension. Patients with a history of alcohol intake or those with viral hepatitis were excluded. Serum samples were collected in fasted state and stored in aliquots at −80 °C until used for analysis. Standard laboratory parameters were evaluated by the central laboratory of the UHE.

All experimental procedures and analyses are described in the supplementary material section, http://links.lww.com/CTG/A215.

## RESULTS

### Characteristics of the study cohort

Eighty-seven subjects, including healthy controls (H, n = 20), NASH-non-HCC without (N, n = 23) or with cirrhosis (Nc, n = 11), and NASH-HCC without (NH, n = 14) or with cirrhosis (NHc, n = 19) were recruited. Controls were younger than patients with a mean BMI of 23.3 kg/m^2^, whereas the majority of NASH-non-HCC and NASH-HCC patients were overweight or obese. Demographic data of individual groups are depicted in Table S1, Supplementary Digital Content 1, http://links.lww.com/CTG/A215.

### Distinctive patterns of liver injury markers and fibrosis in NASH with and without HCC

**Figure 1.**
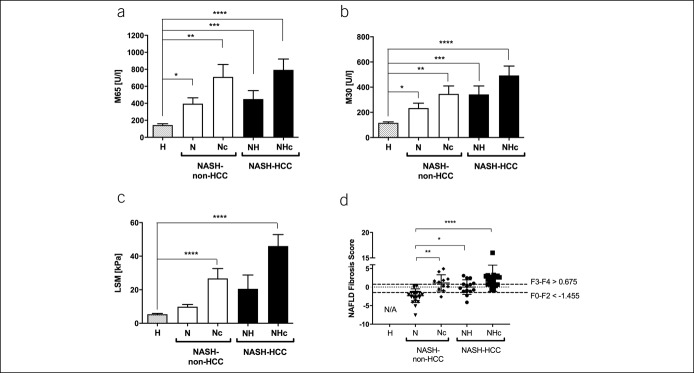
Severity of the disease is associated with cell death, cirrhosis, and increased bile acid levels. Serum levels of cell death marker M65 (**a**) and apoptosis marker M30 (**b**) were increased stepwise in NASH-non-HCC and NASH-HCC as compared to controls. Fibrosis as assessed by liver stiffness measurement (LSM) by transient elastography/FibroScan (**c**) and NAFLD fibrosis score (**d**) reveal higher values in NASH-non-HCC and NASH-HCC while of course those individuals with biopsy proven cirrhosis showed higher levels. In patients with an NAFLD fibrosis score above 0.675, the presence of advanced liver fibrosis can be diagnosed with high accuracy. In patients with an NAFLD fibrosis score below −1.455, advanced liver fibrosis can be excluded with high accuracy. Scores between −1.455 and 0.676 are considered “indeterminate” (21). Data are presented as mean ± SEM. *P < 0.05, **P < 0.01, ***P < 0.001, ****P < 0.0001. NAFLD, nonalcoholic fatty liver disease; NASH-HCC, nonalcoholic steatohepatitis-hepatocellular carcinoma.

Increased levels of ALT, AST, AP, and γGT were observed in NASH-non-HCC and NASH-HCC vs controls (see Table S1, Supplementary Digital Content 1, http://links.lww.com/CTG/A215). Similar to transaminases, in NASH-non-HCC, IL-6 levels, as a marker of hepatic inflammatory response, were higher in noncirrhotic patients and further increased in NHc (see Figure S1A, Supplementary Digital Content 1, http://links.lww.com/CTG/A212). We then assessed the serum levels of 2 distinct cytokeratin-18 fragments, the cell death marker M65 and the hepatocellular apoptosis marker M30. Both markers were significantly elevated in NASH-non-HCC and NASH-HCC compared with controls and further increased in cirrhotics (Figure 1a,b).

We combined the FibroScan liver stiffness measurement (LSM) with the controlled attenuation parameter to assess hepatic fat accumulation. The controlled attenuation parameter was significantly higher in NASH-non-HCC and NASH-HCC compared with controls (see Figure S1B, Supplementary Digital Content 1, http://links.lww.com/CTG/A212). We observed an increase of LSM in NASH-non-HCC and NASH-HCC, which was higher in individuals with cirrhosis (Figure 1c). Because assessment of LSM by FibroScan might be compromised in HCC because of tumor stiffness, we also calculated the NAFLD fibrosis score (21). Here, N showed significantly lower values than individuals with cirrhosis or HCC (Nc, NH, and NHc; Figure 1d).

### Association of serum BA with liver injury and advanced fibrosis

**Figure 2.**
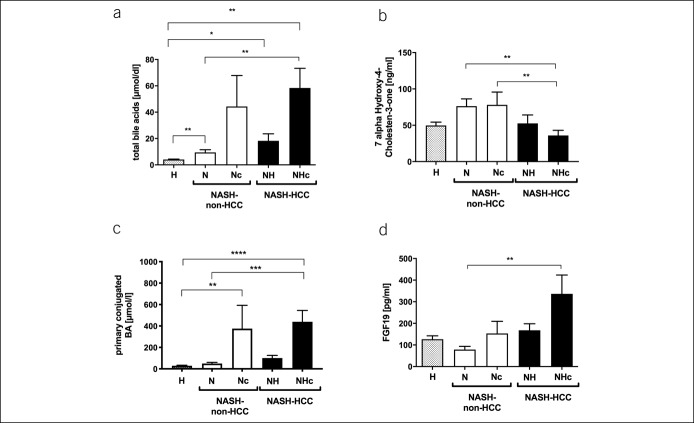
Cirrhosis affects the regulation of bile acid metabolism. Total serum bile acids (**a**) and primary conjugated bile acids (**c**) showed a stepwise increase in NASH-non-HCC and NASH-HCC. Serum levels of 7-alpha-Hydroxy-4-Cholesten-3-one (C4) (**b**) were slightly increased in NASH-non-HCC patients but decreased in NASH-HCC patients. Serum levels of the FXR target molecule FGF19 (**d**) were increased in NASH-HCC patients with cirrhosis. Data are represented as mean ± SEM. *P < 0.05, **P < 0.01, ***P < 0.001, ****P < 0.0001. FXR, farnesoid X receptor; NASH-HCC, non-alcoholic steatohepatitis-hepatocellular carcinoma.

We quantified the total serum BA and detected a significant increase associated with disease severity between healthy, NASH-non-HCC, and NASH-HCC. Furthermore, BA was altered as a function of cirrhosis (Figure 2a). In Nc and NHc, BA was increased compared with noncirrhotic patients. Serum levels of 7α-Hydroxy-4-cholesten-3-one (C4), an intermediate of BA *de novo* synthesis, reflect the activity of the BA synthetic pathway. Although the total BAs were increased in patients, C4-levels were only slightly increased in NASH-non-HCC and even decreased in NASH-HCC, indicating alterations in BA synthesis or export in NASH-HCC (Figure 2b). The total BA was significantly associated with LSM and NAFLD fibrosis score (Table 1). BA levels were also associated with serum markers of liver injury and the tumor marker AFP-L3 (Table 1).

**Table 1.**
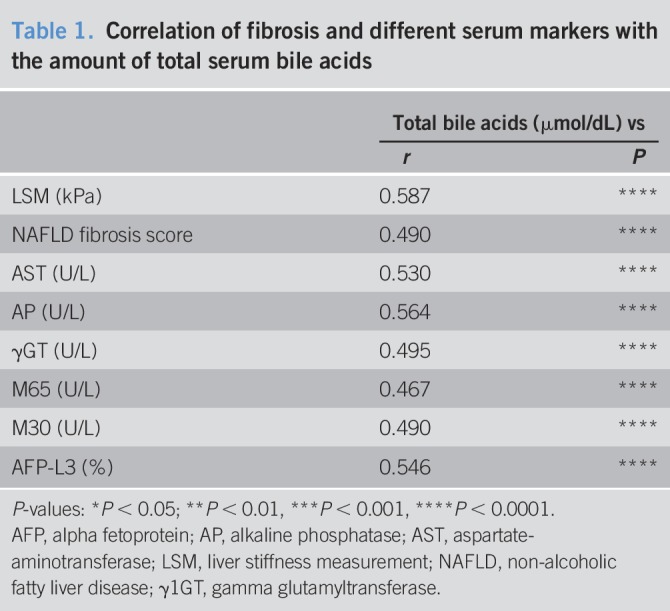
Correlation of fibrosis and different serum markers with the amount of total serum bile acids

### Specific primary conjugated BA is associated with advanced fibrosis

Dysregulation of BA homeostasis and its association with liver injury has previously been described in obese individuals and plays a pivotal role in NAFLD (14). Here, we quantified the serum and fecal concentrations of BA isoforms. Although the total serum BA was elevated in cirrhotic patients, levels of both primary and secondary unconjugated BA were not changed significantly comparing the groups (see Figure S1C,D, Supplementary Digital Content 1, http://links.lww.com/CTG/A212). In line with the abovementioned decrease in C4, serum concentrations of the primary unconjugated BA cholate (CA) appeared lower in NASH-HCC as compared to NASH-non-HCC, whereas chenodeoxycholic acid (CDCA) levels were slightly increased in patients, with even higher levels in Nc and NHc (Table 2). In contrast to secondary conjugated and unconjugated BA, serum concentrations of all conjugated primary BA were increased in NASH-non-HCC and NASH-HCC, with significantly higher levels in Nc and NHc (Table 2, Figure 1c, see Figure 1E, Supplementary Digital Content 1, http://links.lww.com/CTG/A212). Primary conjugated BAs were significantly increased in cirrhotic patients (Figure 1c) and were associated with liver injury and tumor markers (see Table S2, Supplementary Digital Content 1, http://links.lww.com/CTG/A215).

**Table 2.**
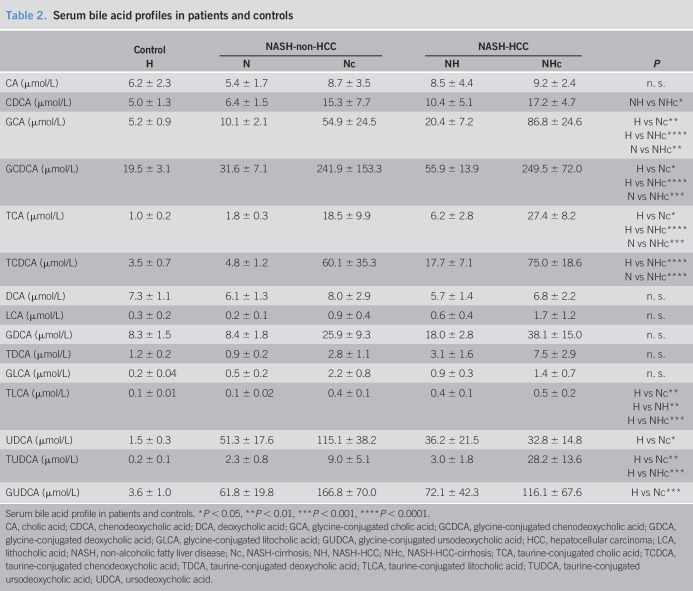
Serum bile acid profiles in patients and controls

In cell culture experiments with primary human HSC treated with primary conjugated BA for 24 hours, we observed an induction of the early profibrotic gene *TGFβ1* (see Figure 2A, Supplementary Digital Content 1, http://links.lww.com/CTG/A213), underlining a pivotal role for primary conjugated BA in fibrogenesis. In contrast to serum levels, fecal BA quantification revealed no significant alterations in the primary conjugated BAs (see Table S3, Supplementary Digital Content 1, http://links.lww.com/CTG/A215).

### FGF19 levels are associated with hepatocellular injury and tumor burden

Intestine-derived FGF19 suppresses the hepatic *de novo* BA synthesis and its expression is controlled by FXR. Serum FGF19 levels were significantly higher in NHc compared with NASH-non-HCC and controls (Figure 2d), whereas C4 levels as intermediate of BA synthesis showed the opposite pattern (Figure 2b). Interestingly, serum levels of the primary unconjugated BA CA and CDCA were again highest in NASH-HCC, indicating mechanisms other than FGF19 involved in the regulation of later BA synthesis steps in NASH-HCC.

We have previously identified adiponectin to interfere with FGF19 signaling because it repressed Cyp7A1 expression *in vivo* and *in vitro* (10). Here, we could show that serum adiponectin was increased in NASH, regardless of HCC occurrence, and an adiponectin/FGF19 ratio paralleled serum C4 levels, indicating a potential synergistic effect in NASH-HCC (see Figure S2B, Supplementary Digital Content 1, http://links.lww.com/CTG/A212). IL-6, which is known to induce adiponectin expression and is a parameter of liver injury and obesity-related systemic inflammation, followed a similar pattern (see Figure S1A, Supplementary Digital Content 1, http://links.lww.com/CTG/A212). IL-6 was associated with the total serum BA (see Figure S2C, Supplementary Digital Content 1, http://links.lww.com/CTG/A213).

As expected, levels of the tumor marker AFP were significantly increased in NASH-HCC (see Figure 2D, Supplementary Digital Content 1, http://links.lww.com/CTG/A213). FGF19 levels were significantly associated with cell death and tumor markers (see Table S3, Supplementary Digital Content 1, http://links.lww.com/CTG/A215).

### Alterations in the gut bacterial community, diversity, and richness in NASH-HCC

Stool samples failed to identify the group-specific microbiota profiles in any of the phylogeny ranks (see Figure S3A, Supplementary Digital Content 1, http://links.lww.com/CTG/A214). In controls, the consistency between bacterial communities converged to higher similarities (see Figure S3B, Supplementary Digital Content 1, http://links.lww.com/CTG/A214). Accordingly, in controls, the bacterial community displayed low variability, with controls showing a similarity in bacterial community of more than 70%. By contrast, patients showed more diverse bacterial community and diminished similarity between individuals.

**Figure 3.**
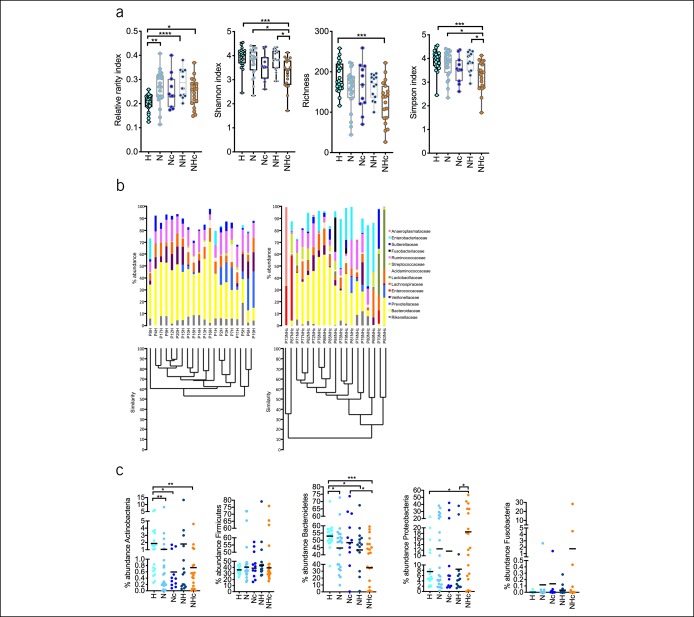
Differences in diversity of bacterial communities in healthy vs NHc patients with distinctive changes on the phylum level. Phylotype richness, Shannon, and Simpson indexes diminished between groups, whereas the relative rarity index increased in N, NH, and NHc as compared to H and shown as median with range (**a**). Comparing healthy individuals and patients (e.g., here, the NHc group) the bacterial community was more homogenous in samples of healthy while NHc showed more diverse bacterial community. Stacked bar plot shows the means in microbial changes of abundances on the family level in H and NHc (**b**). Abundance of Bacteroidetes and Actinobacteria showed decrease from H to NHc, whereas abundances of Firmicutes and Proteobacteria did not reveal differences between the groups (**c**). Relative abundance of Fusobacteria was increased in N, Nc, NH, and NHc because of high abundances in single patients within these groups. Data are represented as median and plotting the individual patients in the graph. *P < 0.05, **P < 0.01, ***P < 0.001, ****P < 0.0001.

The phylotype richness, Shannon, and Simpson indexes as parameters of diversity differed between groups. We observed the most obvious differences between H and NHc. By contrast, the relative rarity index was increased in patients compared with controls (Figure 3a). The results at the family level demonstrated that few bacterial groups comprised the main part of the total community that were uniformly detected in all individuals, whereas in NHc, a higher bacterial community complexity was found (Figure 3c).

### Abundance of individual phylae distinguishes NASH-non-HCC from NASH-HCC

The most abundant phylotypes belonged to the phyla Bacteroidetes, Actinobacteria, Firmicutes, Proteobacteria, and Fusobacteria comprised approximately 99% of the total bacterial community (Figure 3d). Bacteroidetes and to a lesser extent Actinobacteria were gradually decreased in abundance from H to NASH-non-HCC to NASH-HCC. The abundances of Firmicutes and Fusobacteria did not show relevant alterations, whereas high abundance of single phylotypes in 4 individuals significantly affects the abundance of Fusobacteria. The abundance of Proteobacteria was significantly increased in NHc.

### Altered abundance of BA-modifying bacteria in NASH-non-HCC and NASH-HCC

Overall, Bacteroidetes and Firmicutes were the dominant bacterial strains in patients. In NASH-HCC, low abundance of Bacteroidetes promoted the occurrence of smaller bacterial strains.

**Figure 4.**
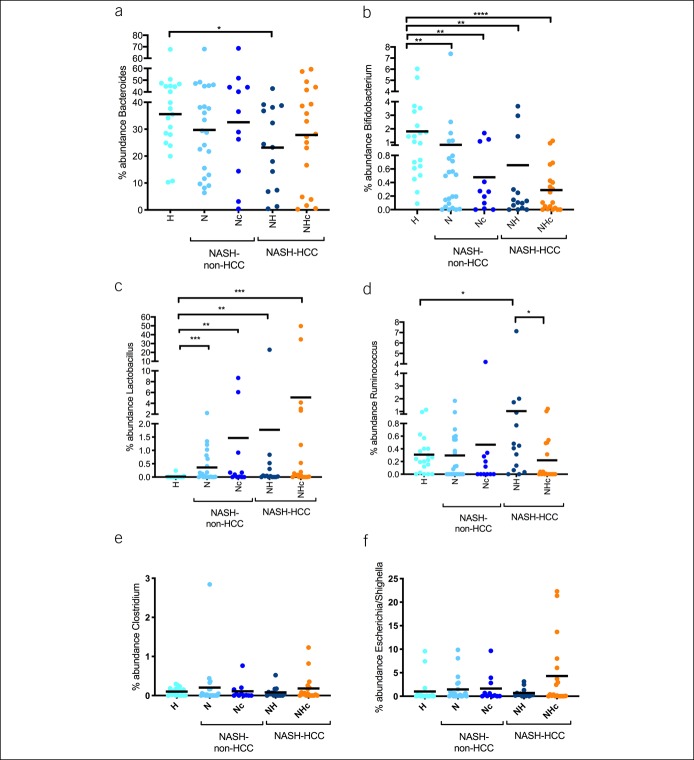
Abundance of bacteria genera that modify bile acids is altered in NASH-non-HCC and NASH-HCC. Relative abundances of Bacteroides (**a**) and Bifidobacterium (**b**) were decreased, whereas Lactobacillus (**c**) and Ruminococcus (**d**) were increased from H to NHc. Relative abundance of Clostridium (**e**) and Escherichia/Shighella (**f**) did not differ between the groups. Data are represented as median and plotting the individual patients in the graph. *P < 0.05, **P < 0.01, ***P < 0.001, ****P < 0.0001. NASH-HCC, nonalcoholic steatohepatitis-hepatocellular carcinoma.

Certain members of bacterial genera are involved in BA deconjugation, oxidation/epimerization, and 7-dehydroxylation (22). Genera that belong to these groups and were identified during the microbiome analysis include Bacteroides, Bifidobacterium, Lactobacillus, Ruminococcus, Clostridium, and Escherichia/Shighella. The abundances of Bacteroides and Bifidobacterium were decreased in NASH-non-HCC and NASH-HCC compared with controls, whereas the abundance of Bacteroides was lowest in NH (Figure 4a,b). Lactobacillus showed a progressive increase in abundance from controls to NHc (Figure 4c). The abundance of Ruminococcus was increased in NH, whereas the abundance of Clostridium and Escherichia/Shighella remained unchanged (Figure 4d–f).

**Figure 5.**
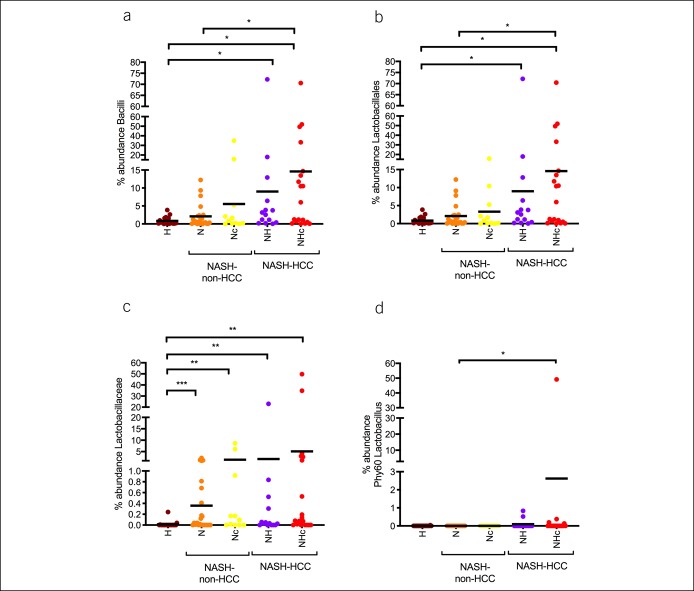
Taxonomic ranks that are related to Lactobacillus showed a progressive increase in abundance from healthy to cirrhotic NASH-HCC patients. The taxonomic ranks class (Bacilli, **a**), order (Lactobacillales, **b**), family (Lactobacillaceae, **c**), and phylotype (Phy60 Lactobacillus, **d**) showed a progressive increase of abundances from control to NHc. Data are represented as median and plotting the individual patients in the graph. *P < 0.05, **P < 0.01, ***P < 0.001, ****P < 0.0001. NASH-HCC, nonalcoholic steatohepatitis-hepatocellular carcinoma.

Interestingly, the analysis of Lactobacillus-related ranks showed a progressive increase in abundance in the ranks class, order, family, and phylotype (Phy60) from controls to NHc (Figure 5). Correlations of individual serum parameters or single BA with changes in the abundance of Lactobacillus showed that total BA, primary conjugated BA and the individual BA glycine-conjugated cholic acid (GCA), glycine-conjugated chenodeoxycholic acid (GCDCA), taurine-conjugated cholic acid (TCA), and taurine-conjugated chenodeoxycholic acid (TCDCA) were associated with the abundance of Lactobacillus (Table 3). Translationally, this abundance was associated with LSM and γGT (Table 3).

**Table 3.**
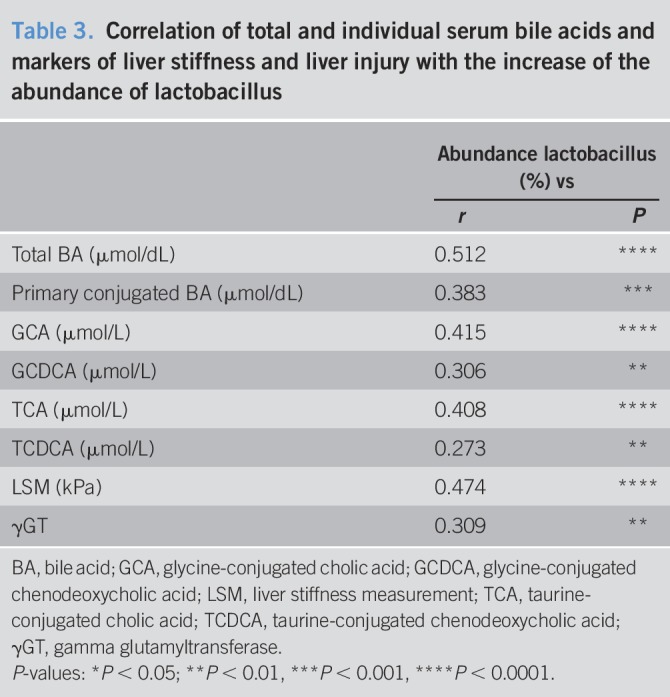
Correlation of total and individual serum bile acids and markers of liver stiffness and liver injury with the increase of the abundance of lactobacillus

## DISCUSSION

Here, we analyzed gut microbiome alterations, BA composition, and serum parameters of the BA metabolism in NASH-HCC. Therefore, we noninvasively assessed liver injury, fibrosis, and BA metabolism, as well as gut microbiome composition in healthy individuals compared with patients with NASH with and without HCC and with and without cirrhosis. Total serum and primary conjugated BAs were clearly associated with advanced fibrosis in NASH-HCC, indicating a potential role in fibrosis-related tumorigenesis (10,12,23). These alterations were accompanied by an increased abundance of several bacterial strains, particularly Lactobacilli and Bacteroides, which predominantly express bile salt hydrolase (BSH), an enzyme involved in deconjugation. Abundance of Lactobacilli was as well associated with liver stiffness and injury and most likely secondary to the high availability of primary conjugated BAs as a substrate (22). In individuals with NASH, Jiao et al. observed a similar shift in abundance of BSH expressing bacteria. Here, an increase in BA levels and an altered composition of the BA pool with an increase in secondary conjugated BA was observed (24). Although we observed a similar trend in our cohort, the increase of secondary conjugated BA was not significant. Given the lack of liver biopsies, it was impossible for us to measure the gene-expression levels of the BA metabolism–related genes (e.g., CYP7A1). However, when assessing the serum C4 levels, we found no evidence of increased BA synthesis. Although C4 levels were increased in NASH non-HCC, they did not correlate with the overall BA levels in our cohort. Interestingly, Jiao et al. reported that FGF19 was lower in a cohort of patients with NASH, but no distinction was made between cirrhosis and noncirrhosis in this study. In our cohort, the FGF19 levels were higher in cirrhotic patients, but we did not observe significant associations with other parameters of BA metabolism.

By contrast, as an important mediator of BA metabolism and key messenger between the gut and liver, elevated FGF19 levels were associated with tumor and cell-death markers in HCC, independent of fibrosis assessment. This interrelation affects the early steps of primary hepatic BA synthesis while serum adipokines and inflammation appear to further modify primary BA synthesis in NASH-HCC. This finding is of particular interest because FGF19 expression is known to correlate with tumor progression and poorer prognosis in HCC and is associated with wnt/β-catenin signaling (25,26).

Gut-liver interactions as well as diet-induced alterations in gut and metabolic homeostasis are increasingly implicated in NASH and NASH-HCC. Distinct changes or shifts in the composition of intestinal bacteria have been described for several metabolic and inflammatory diseases (27–29). The effects of the gut microbiome on liver inflammation exceed the role of bacteria-derived toxins and lipopolysaccharides. In fact, the metabolism of distinct bacterial groups affects the mucosal barrier, hepatic inflammation, fibrogenesis, and tumorigenesis (30,31). Gut microbiota have an impact on energy balance, altering the uptake of calories derived from food or alcohol (32). Emerging data indicate that certain characteristic changes in the gut microbiome are associated with NAFLD and cirrhosis (14,29,48,49). As mentioned above, we identified the abundance of the BA deconjugating Lactobacilli in patients with NASH and NASH-HCC with advanced fibrosis. In obese children, an increased abundance of Lactobacillus strains was associated with NAFLD and NASH (33). Although the biological function remains unclear, several publications indicate a beneficial effect of these strains. In colon cancer, increased Lactobacillus abundance was related to antitumor effects (34,35). Beyond these observations, we did not detect a more prominent effect on the microbiome because of cirrhosis nor a specific signature for NASH cirrhosis (49).

An altered composition of the individual BA pool may trigger hepatic inflammation during NAFLD progression and thus, promote HCC-tumorigenesis. We previously demonstrated that in morbidly obese patients with NASH, BA levels were increased compared with milder stages of NAFLD (10). This was recently confirmed by Puri et al., (12) who found that altered circulating BA levels correlate with the severity and progression of NAFLD, and abundance of conjugated cholate was associated with advanced fibrosis. Primary BAs are synthesized in hepatocytes from cholesterol and after conjugation to glycine or taurine are excreted from the liver. Gut bacteria affect the BA composition, as they convert primary to secondary BA via dehydroxylation and deconjugation. There are specific groups of BA-modifying bacterial strains that are involved in converting primary BA to different secondary BA finally forming the complex BA pool (22). We here identified specific alteration of abundance of BA converting strains, which correlate with total and primary conjugated BA. In line with our observations, Ponziani et al. correlated changes within the gut microbiome with an influence on inflammation and hepatocarcinogenesis in cirrhotic HCC patients compared with noncirrhotic HCC patients. Here, among other strains, abundance of Bacteroides was also increased in HCC (29). The composition of the intestinal microbiome, therefore, also affects the composition of BA pool and *vice versa*. Dysbiosis in the fecal microbiome composition alters the composition of the BA pool and has been described in NAFLD (14) or NASH (24, Jiao). Hepatocellular BA accumulation causes cytotoxicity and leads to mitochondrial stress, triggers cell death, inflammation, and hepatocarcinogenesis (17,36). The cytotoxic effect of BA is dependent of the composition of the BA pool, where hydrophobic BA exhibit more toxicity (37). More specifically, dysbiosis-associated gut microbial BA metabolites such as DCA have been linked to HSC senescence and hepatic carcinogenesis. Conversely, modulation of the gut microbiome by antibiotic therapy reduced hepatic carcinogenesis in a high fat diet mouse model of NASH, which was associated with a reduction in DCA (17). However, in this cohort, we did not observe such an increase in distinct “toxic” BA.

Furthermore, levels of hydrophobic BA such as TCDCA and TLCA are altered in high fat diet mouse models and may contribute to carcinogenesis in NASH by promoting cell proliferation and inflammation (38). In a rodent model of NASH, BA and cholesterol levels were increased and manipulation of the microbiome by antibiotic treatment reduced the accumulation of secondary BA (39). Other studies demonstrated that levels of TCA, TCDCA, GCA, and GCDCA were significantly increased in patients with cirrhosis and NASH-HCC (40,41). In our cohort, total and primary conjugated BAs were increased in non-HCC and HCC with higher levels in cirrhotics. Furthermore, total and primary conjugated BAs are associated with increased fibrosis, liver injury, and tumor markers. Individual BAs have previously been described to induce HSC proliferation, which is an essential and important mechanism of fibrogenesis (42) and indeed, here we found GCA-, TCA-, GCDCA-, and TCDCA-induced *TGFβ1* expression in HSC *in vitro*. We thus propose that higher levels of individual BA, and especially primary conjugated BA, are associated with HSC activation and fibrogenesis in cirrhosis, and this might contribute to tumorigenesis *via* induction of fibrosis.

The main molecule connecting BA homeostasis in the gut liver axis is the FXR target molecule FGF19. Enterocyte-derived FXR is activated by BA and regulates hepatic *de novo* primary BA synthesis via excretion of FGF19. FGF19 is increased in HCC and associated with a poor prognosis (43). As a consequence, FGF19 is considered as a promising target in HCC therapy (44,45). BA and synthetic FXR agonists are used to prevent BA overload and cholestatic hepatocellular injury in NAFLD (46). Although UDCA reduced FGF19 levels (47), OCA and other FXR agonists tested in the treatment for NASH, induced FGF19 levels (48). The FGFR4-Klothoβ signaling pathway plays a pivotal role as a driver in certain subtypes of HCC. FGF401 is a highly selective FGFR4 antagonist and preliminary data of a recent clinical trial showed promising clinical effects in patients with HCC (49). We found that FGF19 levels were elevated in NASH-HCC but not in NASH with highest levels in NHc. In the HCC cohort, serum levels of AFP were positively correlated to FGF-19, indicating a potential role of the FGF19/FGFR4 pathway in hepatocarcinogenesis. In line with our previous observations and data derived from recent publications, we assume an induction of BA synthesis in advanced NASH, which might be attributed to alterations in death receptor expression and adipokine signaling, affecting the FGF19/FGFR4 pathway (10,12). However, we observed a discrepancy between serum levels of the BA synthesis intermediate C4 and serum levels of primary unconjugated BAs indicating further alterations in later steps of hepatic BA synthesis or export, which might be related to alterations in serum adiponectin or IL-6 in NASH-HCC. Probiotic treatment may reduce BA levels via the FGF19 axis (50,51). In our study, FGF19 levels were highest in NHc but the increase of FGF19 was more likely related to carcinogenesis than to cirrhosis.

Although we observed striking alterations in FGF19 signaling, associated with NASH-HCC on the one hand and significant changes in BA homeostasis associated with fibrosis on the other hand, it remains unclear whether these mechanisms occur independently or synergistically during hepatocarcinogenesis. Another limiting factor of this study is that the dietary fat intake was neglected as an influencing factor both on the BA metabolism and on the development and the progress of NASH. Based on our findings, further analysis of this interaction is needed in future studies, including data from multicenter studies and generation of longitudinal data to develop potential therapeutic options based on targeted modifications of the BA profile, dietary factors, and the gut microbiome.

## CONFLICTS OF INTEREST

**Guarantor of the article:** Lars P. Bechmann, MD, MBA.

**Specific author contributions:** S.S. (acquisition, analysis and interpretation of data; study design; technical support; statistical analysis; drafting of the manuscript), J.B. (acquisition, analysis and interpretation of data; study design; statistical analysis; drafting of the manuscript; study supervision), I.M. (data acquisition, analysis and interpretation of data; statistical analysis), P.M. (acquisition, analysis and interpretation of data; statistical analysis), R.V.-V. (acquisition and analysis of microbiome data; statistical analysis, drafting of the manuscript), S.B. (acquisition and analysis of data for BAs profile; technical support), C.L. (measurement of bile acid profiles; technical support), A.W. (data acquisition), C.W. (data acquisition), S.A. (data acquisition), S.H. (acquisition and analysis of data for C4 quantification; technical support), A.L. (critical revision of the manuscript for important intellectual content), K.N.F. (critical revision of the manuscript for important intellectual content; technical support), H.M. (critical revision of the manuscript for important intellectual content; technical support), F.J.C. (critical revision of the manuscript for important intellectual content), S.L.F. (critical revision of the manuscript for important intellectual content), G.G. (obtained funding; critical revision of the manuscript for important intellectual content), M.T. (critical revision of the manuscript for important intellectual content), A.C. (obtained funding; drafting of the manuscript; critical revision of the manuscript for important intellectual content), L.P.B. (study concept and design; acquisition, analysis and interpretation of data; statistical analysis; study supervision; drafting of the manuscript; obtained funding).

**Financial support:** This work was supported by the German Research Foundation (DFG; project number BE-3967/3-1; L.P.B.), Dr Werner Jackstaedt Foundation (L.P.B., J.B.), the European association for the study of the liver (EASL; Short-term fellowship; S.S.), *Deutsche Leberstiftung* (*Vernetzungsstipendium*; S.S.), SAF2017-87919-R, EXOHEP-CM S2017/BMD-3727, NanoLiver-CM Y2018/NMT-4949, ERAB Ref. EA 18/14, AMMF 2018/117, UCM-25-2019 and COST Action CA17112. F.J.C. is a Ramón y Cajal Researchers RYC-2014-15242 and a Gilead Liver Research Scholar 2018, NIHRO1DK56621 (S.L.F.), US Department of Defense (CA150272; S.L.F.), 1P30CA 196521-01 (S.L.F.), Wilhelm-Laupitz Foundation (A.C.).

**Potential competing interests:** None to report.

**Study Highlights**

**WHAT IS KNOWN**

✓ Serum bile acid levels are increased in subjects with NAFLD and are associated with disease severity.
✓ Alterations in gut microbiota were previously identified as important mediators in disease progression of NAFLD and NASH.
✓ These alterations are associated with accumulation of specific “toxic” metabolites involved in hepatic inflammation and carcinogenesis.

**WHAT IS NEW HERE**

✓ We present data linking the interaction of liver and gut in NASH-related HCC in the absence or presence of cirrhosis.
✓ FGF19 levels were associated with attenuation of early steps of hepatic BA synthesis, advanced tumor burden and liver injury in NASH-HCC, independent of liver fibrosis. Hepatic BA synthesis or export appears to be further affected by adipocytokines and systemic inflammation.
✓ In NASH-HCC, we identified specific alterations in gut microbiota associated with BA metabolism, specifically the availability of substrates for biles salt hydrolase, an enzyme involved in BA deconjugation. These alterations were associated with hepatic fibrosis and injury.

**TRANSLATIONAL IMPACT**

✓ While FGF19 is associated with tumorigenesis in NASH-HCC, independent of fibrosis, cirrhosis-specific alterations in primary unconjugated BAs in NASH-HCC and the associated abundance of gut bacteria indicate two distinct mechanisms in hepatocarcinogenesis and thus represent potential targets for therapeutic approaches.

## Supplementary Material

SUPPLEMENTARY MATERIAL
