# Supplementary material for: Altered Microbiota Diversity and Bile Acid Signaling in Cirrhotic and Noncirrhotic NASH-HCC
Source: Clin Transl Gastroenterol. 2020 Mar 4;11(3):e00131. doi: 10.14309/ctg.0000000000000131 (PMC7145043; doi:10.14309/ctg.0000000000000131)
Supplement: SUPPLEMENTARY MATERIAL [file ct9-11-e00131-s001.docx]

**Supplementary Methods**

*Quantification of 7 alpha-hydroxy-4-cholesten-3-one (C4)*

7 alpha-hydroxy-4-cholesten-3-one (C4) was determined via HPLC as described by Axelson et al(1), using 7β-hydroxy-4-cholesten-3-one as internal standard(2). In brief, 100 ng of the internal standard 7β-hydroxy-4-cholesten-3-one (Steraloids, Newport, RI, USA) were added to each sample. Samples were sonicated in a water bath (35 kHz, 160 W) for 20 min and loaded on prewashed jacketed glass columns using octadecylsilane-bonded silica (Preparative C18, 125 Å, 55-105 μm; Waters, MI, USA). After washing the columns, C4 was eluted with hexane-chloroform (75:25, v/v, LiChrosolv; Merck and Rotisolv high-performance liquid chromatography [HPLC]; Carl Roth, Karlsruhe, Germany). The samples were dried under nitrogen and reconstituted in methanol. For HPLC, acetonitril/water (97.5:2.5 v/v, LiChrosolv; Merck) served as the mobile phase on a heated (64°C) reversed phase silica Nova-Pak C18 column (3.9×300 mm, 4 μm particle size) with an upstream guard column (Nova-Pak C18 3.9×20 mm, 4 μm particle size, Waters, Milford, MA, USA). C4 was detected by an ultraviolet-detector at wavelength 241 nm and an integrator (CR-6A; Shimadzu) and was calculated from the area under the peak, corrected for the amount recovered internal standard.

*Fecal samples and microbiome analysis*

Fecal samples were collected from every patient in sterile tubes and stored at -80°C until to DNA isolation. Individuals were excluded if they received antibiotic treatment during the past 4 weeks. DNA was isolated using the QIamp-DNA isolation kit following manufacturer’s instructions (Qiagen, Hilden, Germany) including a mechanical lysis step using dry bead tubes (MoBio Laboratories Inc., Carlsbad, CA, USA) and the Fast Prep™-24 instrument (MP Biomedicas, Solon, OH, USA) at 6.0 m/s for 45sec (two times). Amplicon libraries were generated as previously described(3) and sequenced on a MiSeq (2×250 bp, Illumina, Hayward, CA, USA). All FastaQ files were analyzed using dada2 package in R (www.r-project.org) and as result a unique table containing all samples with the sequence reads and abundances was generated. All samples were resampled to equal the smallest library size of 12,448 reads using the phyloseq package and returning 5,205 phylotypes. Sequence reads were assigned to a taxonomic affiliation based on the naïve Bayesian classification with a pseudo-bootstrap threshold of 80%. Relative abundances, in percentage of Phylotypes, Genus, Family, Order, Class and Phylum were used for downstream analyses. The vegan package (http://CRAN.R-project.org/package=vegan) was used to generate rarefaction curves, the EcoIndR for calculating the richness, relative rarity, Shannon, Simpson, and taxonomy diversity indices. For microbiome analysis both packages from R were used (version 3.4.2. 2017). Multivariate analyses were performed with Past3 (https://folk.uio.no/ohammer/past/) and univariate analyses were performed with GraphPad Prism 7 (GraphPad Software Inc., San Diego, CA, USA). The data comprising the six taxonomy ranks were used to construct sample-similarity matrices using the Bray-Curtis algorithm, where samples were clustered with 1,000 bootstrap. Significant differences between a priori predefined groups of specimens were evaluated using analysis of similarity (ANOSIM with 9.999 permutations) and Permutational multivariate analysis of variance (PERMANOVA with 9.999 permutations) and groups were considered significantly different if the p value was ≤0.05. The abundances of the six taxonomy ranks of those phylotypes with a mean >1%, were compared by the Mann-Whitney test using the package exactRankTests (CIs at 95%) from R.

Bacterial diversity in feces was compared between groups in order to identify individual bacteria as well as disease-associated bacterial signatures. Detected phylotypes were taxonomically assigned to 11 phyla, 23 classes, 37 orders, 74 families and 151 genera. The global bacterial profiles at all phylogeny ranks (Phylotypes, Genus, Family, Order, Class and Phylum) were compared using group-average agglomerative hierarchical clustering following sample-pairwise comparisons.

*Serum and fecal sample BA profiling*

Fecal BA were extracted by sonification in 1:3 diluted extraction buffer (ethanol and phosphate buffer; Sigma-Aldrich, Steinheim, Germany) from approx. 300mg of feces; supernatants were used for quantification. Quantification of primary and secondary BA in serum or from fecal sample extracts was performed by Liquid Chromatography coupled to Electrospray Ionization Tandem Mass Spectrometry (LC-ESI-MS/MS) using the Biocrates® Bile Acids Kit (BIOCRATES Life Sciences AG, Innsbruck, Austria) which covers 16 individual BA. Data analysis was completed using the Biocrates MetIDQ software(4).

*ELISA*

Serum levels of the overall cell death marker M65, and apoptosis marker M30 were measured using commercially available kits from TecoMedical (Sissach, Switzerland). FGF19 serum levels were quantified using the Quantikine ELISA-kit from R&D Systems (Minneapolis, MN, USA).

*Bioinformatics and statistical analysis*

Statistical significance was determined using an unpaired, two-tailed t-test or by one-way ANOVA and nonparametric test (Kruskal-Wallis and Dunn’s multiple comparison test correction for individual experimental conditions). Correlation analysis was performed using linear regression analysis; all analyses were performed with GraphPad Prism 7. If not stated otherwise all data are presented as mean ± SEM. Significance was assumed at p ≤0.05.

*Isolation and cultivation of human primary hepatic stellate cells*

Primary human hepatic stellate cells were isolated as described previously *(5)* and cultivated using Stellate Cell Medium (ScienCell, Carlsbad, CA, USA) supplemented with supplied 10% FBS, stellate cell growth supplement, 1000U/ml penicillin, and 0.1mg/ml streptomycin in an atmosphere with 5% CO_2_ under 37°C following standard protocols.. The liver specimens for cell isolation were obtained from fresh tumor resections. All patients provided written documentation of informed consent. Ethics Committee (Institutional Review Board) of the Otto-von-Guericke-University approved the study (reference number: 208/17). The study protocol conformed to the ethical guidelines of the declaration of Helsinki.

*Cell culture experiments using human primary hepatic stellate cells*

Following isolation, 1x10^6^/cm^2^ cells were seeded onto culture plates and on the next day treated with specific BA (Sigma-Aldrich). Final concentration of 50µM was adjusted by dilution in culture medium. Cells were incubated for 24h and washed with PBS prior to RNA-isolation using the RNeasy Mini Kit (Qiagen).

*Quantitative real time PCR*

Reverse transcription was performed with the QuantiTect-RT kit (Qiagen) using 1µg of total RNA. Specific mRNA expression levels of TGFβ (forward primer: 5´-gtacctgaacccgtgttgct-3´; reverse primer: 5´-gaacccgttgatgtccactt-3´) were measured by quantitative real time PCR (qRT-PCR) performed on a CFX96 Touch Real-Time PCR Detection System (Bio-Rad, Munich, Germany) using QuantiTect SYBR-Green Kit (Qiagen) in a volume of 15μl including 2μl of cDNA. Melting curves were collected to ascertain specificity of PCR products. Changes in mRNA expression were calculated by the ΔΔ-Ct method and are presented as foldchange in relation to the reference gene HPRT (forward primer: 5´-gaccagtcaacaggggacat-3´; reverse primer: 5´-cttgcgaccttgaccatctt-3´).

**Supplementary Table S1:** Patient demographics and serum parameters of liver injury. Individual p-values are stated in case of significant differences. If not included, p-value is non-significant.

|  | **Control** | **NASH-Non-HCC** | | **NASH-HCC** | | **p-value** |
| --- | --- | --- | --- | --- | --- | --- |
|  | **H** | **N** | **Nc** | **NH** | **NHc** |  |
| **Cirrhosis** | No (n=20)  m=12/f=8 | No (n=23)  m=11/f=12 | Yes (n=11)  m=10/f=1 | No (n=14)  m=12/f=4 | Yes (n=19)  m=16/f=3 |  |
| **Dyslipidemia** | 0 % | 35.5% | 62.5 % | 50 % | 41.2 % |  |
| **Diabetes mellitus type 2** | 0 % | 43.5 % | 81.8 % | 57.1 % | 68.4 % |  |
| **Arterial hypertension** | 0 % | 43.5 % | 72.3 % | 92.9 % | 84.2 % |  |
| **Metabolic syndrome** | 0 % | 55.6 % | 77.8 % | 83.3% | 77.8 % |  |
| **Age (years)** | 23.3 ± 2.8 | 49.0 ± 2.8 | 60.2 ± 1.8 | 71.5 ± 3.1 | 68.6 ± 2.1 | H vs. N *  H vs. Nc ***  H vs. NH ****  H vs. NHc ****  N vs. NH **  N vs. NHc ** |
| **BMI (kg/m^2^)** | 23.3 ± 2.5 | 29.8 ± 1.2 | 32.5 ± 2.3 | 29.3 ± 1.5 | 30.8 ± 1.3 | H vs. N ****  H vs. Nc ****  H vs. NH **  H vs. NHc **** |
| **AST (U/l)** | 25.9 ± 3.0 | 55.6 ± 10.6 | 50.0 ± 5.2 | 66.6 ± 14.6 | 93.6 ± 25.2 | H vs. N *  H vs. Nc **  H vs. NH **  H vs. NHc **** |
| **ALT (U/l)** | 25.1 ± 3.3 | 72.6 ± 13.2 | 50.0 ± 8.8 | 63.7 ± 22.9 | 48.9 ± 4.7 | H vs. N ***  H vs. Nc *  H vs. NHc ** |
| **AP (U/l)** | 56.6 ± 3.7 | 105.0 ± 12.1 | 117.7 ± 12.5 | 201.9 ± 57.1 | 213.4 ± 72.9 | H vs. N **  H vs. Nc ***  H vs. NH ****  H vs. NHc **** |
| **γGT (U/l)** | 17.7 ± 1.8 | 100.3 ± 21.6 | 172.1 ± 32.8 | 201.6 ± 49.0 | 271.6 ± 34.5 | H vs. N **  H vs. Nc ****  H vs. NH ****  H vs. NHc ****  N vs. NHc ** |

**Supplementary Table S2:** Correlation of Serum parameters with primary conjugated bile acids. P-values; *p<0.05; **p<0.01, ***p<0.001, ****p<0.0001

|  | **Primary conjugated bile acids vs.** | |
| --- | --- | --- |
|  | **r** | **p-value** |
| **M65** | 0.38 | *** |
| **M30** | 0.43 | **** |
| **AST** | 0.26 | * |
| **AP** | 0.42 | **** |
| **γGT** | 0.40 | *** |
| **AFP** | 0.32 | ** |
| **AFP-L3** | 0.50 | **** |

**Supplementary Table S3:** Faecal bile acid profile in patients and controls.

|  | **Control** | **NASH-Non-HCC** | | **NASH-HCC** | | **p-value** |
| --- | --- | --- | --- | --- | --- | --- |
|  | **H** | **N** | **Nc** | **NH** | **NHc** |  |
| CA (µmol/g) | 0.30 ± 0.11 | 3.81 ± 1.46 | 2.02 ± 1.11 | 0.16 ± 0.06 | 2.70 ± 1.20 | n. s. |
| CDCA (µmol/g) | 0.40 0.17 | 1.98 ± 0.61 | 1.68 ± 0.74 | 0.44 ± 0.19 | 1.97 ± 0.94 | n. s. |
| GCA (µmol/g) | 0.013 ± 0.003 | 0.03 ± 0.008 | 0.014 ± 0.006 | 0.02 ± 0.008 | 0.06 ± 0.03 | n. s. |
| GCDCA (µmol/g) | 0.015 ± 0.003 | 0.02 ± 0.01 | 0.013 ± 0.004 | 0.015 ± 0.004 | 0.06 ± 0.03 | n. s. |
| TCA (µmol/g) | 0.006 ± 0.002 | 0.14 ± 0.11 | 0.002 ± 0.0005 | 0.003 ± 0.001 | 0.03 ± 0.022 | n. s. |
| TCDCA (µmol/g) | 0.006 ± 0.002 | 0.007 ± 0.003 | 0.002 ± 0.0005 | 0.005 ± 0.003 | 0.08 ± 0.07 | n. s. |
| DCA (µmol/g) | 8.70 ± 1.04 | 7.86 ± 1.17 | 7.16 ± 1.63 | 8.06 ± 1.03 | 4.46 ± 1.24 | n. s. |
| LCA (µmol/g) | 7.69 ± 1.09 | 7.84 ± 1.46 | 8.22 ± 1.89 | 11.16 ± 2.48 | 2.24 ± 1.78 | n. s. |
| GDCA (µmol/g) | 0.08 ± 0.03 | 0.04 ± 0.01 | 0.06 ± 0.02 | 0.05 ± 0.01 | 0.03 ± 0.01 | n. s. |
| TDCA (µmol/g) | 0.02 ± 0.009 | 0.01 ± 0.008 | 0.01 ± 0.006 | 0.01 ± 0.003 | 0.01 ± 0.005 | n. s. |
| GLCA (µmol/g) | 0.02 ± 0.004 | 0.01 ± 0.004 | 0.02 ± 0.007 | 0.03 ± 0.01 | 0.01 ± 0.004 | n. s. |
| TLCA (µmol/g) | 0.01 ± 0.005 | 0.01 ± 0.002 | 0.01 ± 0.003 | 0.01 ± 0.003 | 0.01 ± 0.008 | n. s. |
| UDCA (µmol/g) | 0.16 ± 0.05 | 3.10 ± 0.91 | 3.61 ± 1.21 | 1.25 ± 0.73 | 0.93 ± 0.39 | H vs. Nc * |
| TUDCA (µmol/g) | 0.001 ± 0.0003 | 0.02 ± 0.01 | 0.05 ± 0.05 | 0.003 ± 0.002 | 0.03 ± 0.02 | n. s. |
| GUDCA (µmol/g) | 0.002 ± 0.0006 | 0.11 ± 0.06 | 0.04 ± 0.02 | 0.03 ± 0.01 | 0.09 ± 0.06 | n. s. |

**Supplementary Table S4:** Correlation of different cell death and tumor serum markers with FGF19 serum levels. P-values; *p<0.05; **p<0.01, ***p<0.001, ****p<0.0001.

|  | **FGF19 [pg/ml] vs.** | |
| --- | --- | --- |
|  | **r** | **p-value** |
| **M65 [U/l]** | 0.424 | ** |
| **M30 [U/l]** | 0.315 | *** |
| **AFP [U/ml]** | 0.545 | **** |
| **AFP-L3 [%]** | 0.506 | **** |
| **DCP [ng/ml]** | 0.346 | ** |

**Supplementary Figure Legends**

**Supplementary Figure S1: Steatosis as well as primary and secondary unconjugated BA were not changed significantly.** Serum levels of the proinflammatory cytokine IL-6 were increased in N and NHc as compared to controls (A). In comparison to H**,** NASH and NASH-HCC patients showed higher levels of steatosis as measured by CAP (controlled attenuation parameter; B) while this difference was not significant. Total primary unconjugated (C) and total secondary unconjugated (D) bile acids were not changed comparing the different groups. Serum levels of secondary conjugated bile acids were not changed significantly between the different groups (E). Data are presented as mean ± SEM. * p<0.05, ** p<0.01, *** p<0.001, ****p<0.0001.

**Supplementary Figure S2: Different primary conjugated BA have an impact on pro-fibrogenic gene expression in primary human hepatic stellate cells.** Expression levels of *TGFβ* (transforming growth factor beta) were increased in primary human hepatic stellate cells treated with the single primary conjugated BA GCA (glycocholic acid), TCA (taurocholic acid), GCDCA (glycochenodeoxycholic acid), or TCDCA (taurochenodeoxycholic acid) in a concentration of 50µM for 24h (A). Serum levels of Adiponectin were increased in patients with fibrosis (B) and IL-6 serum levels are related to increased total serum BA levels (C). Serum levels of the tumor marker AFP-L3 showed an increase in patients with NASH-HCC as expected (D). Data are represented as mean ± SEM. * p<0.05, ** p<0.01, *** p<0.001, ****p<0.0001.

**Supplementary Figure S3: Individual samples for microbiome analysis do not show group clustering and the diversity of bacterial communities in healthy versus NHc patients are different comparing groups.** Following group average agglomerative hierarchical clustering and pairwise comparison of faecal samples for microbiome analysis did not show distinct clusters of the defined groups in any of the phylogeny ranks (A). Formal pair-wise comparisons between the global bacterial assemblages revealed that the global bacterial structures of H/Nc, H/NH and H/NHc were significantly different at all taxonomy ranks (B). Comparing healthy individuals and patients (e.g. here NHc group) the bacterial community was more homogenous in samples of healthy while NHc showed more diverse bacterial community (B).

**Supplementary References**

1. Axelson M, Aly A, Sjövall J. Levels of 7 alpha-hydroxy-4-cholesten-3-one in plasma reflect rates of bile acid synthesis in man. FEBS Lett. 1988;239:324–328.

2. Sauter GH, Münzing W, Ritter C von, *et al.* Bile acid malabsorption as a cause of chronic diarrhea: diagnostic value of 7alpha-hydroxy-4-cholesten-3-one in serum. Dig. Dis. Sci. 1999;44:14–19.

3. Rath S, Heidrich B, Pieper DH, *et al.* Uncovering the trimethylamine-producing bacteria of the human gut microbiota. Microbiome 2017;5:54.

4. Pham HT, Arnhard K, Asad YJ, *et al.* Inter-Laboratory Robustness of Next-Generation Bile Acid Study in Mice and Humans: International Ring Trial Involving 12 Laboratories. The Journal of Applied Laboratory Medicine 2016;1:129–142.

5. Werner M, Driftmann S, Kleinehr K, *et al.* All-In-One: Advanced preparation of Human Parenchymal and Non-Parenchymal Liver Cells. PLoS ONE 2015;10:e0138655.
