# Supplementary figures and images for: Altered Microbiota Diversity and Bile Acid Signaling in Cirrhotic and Noncirrhotic NASH-HCC
Source: Clin Transl Gastroenterol. 2020 Mar 4;11(3):e00131. doi: 10.14309/ctg.0000000000000131 (PMC7145043; doi:10.14309/ctg.0000000000000131)

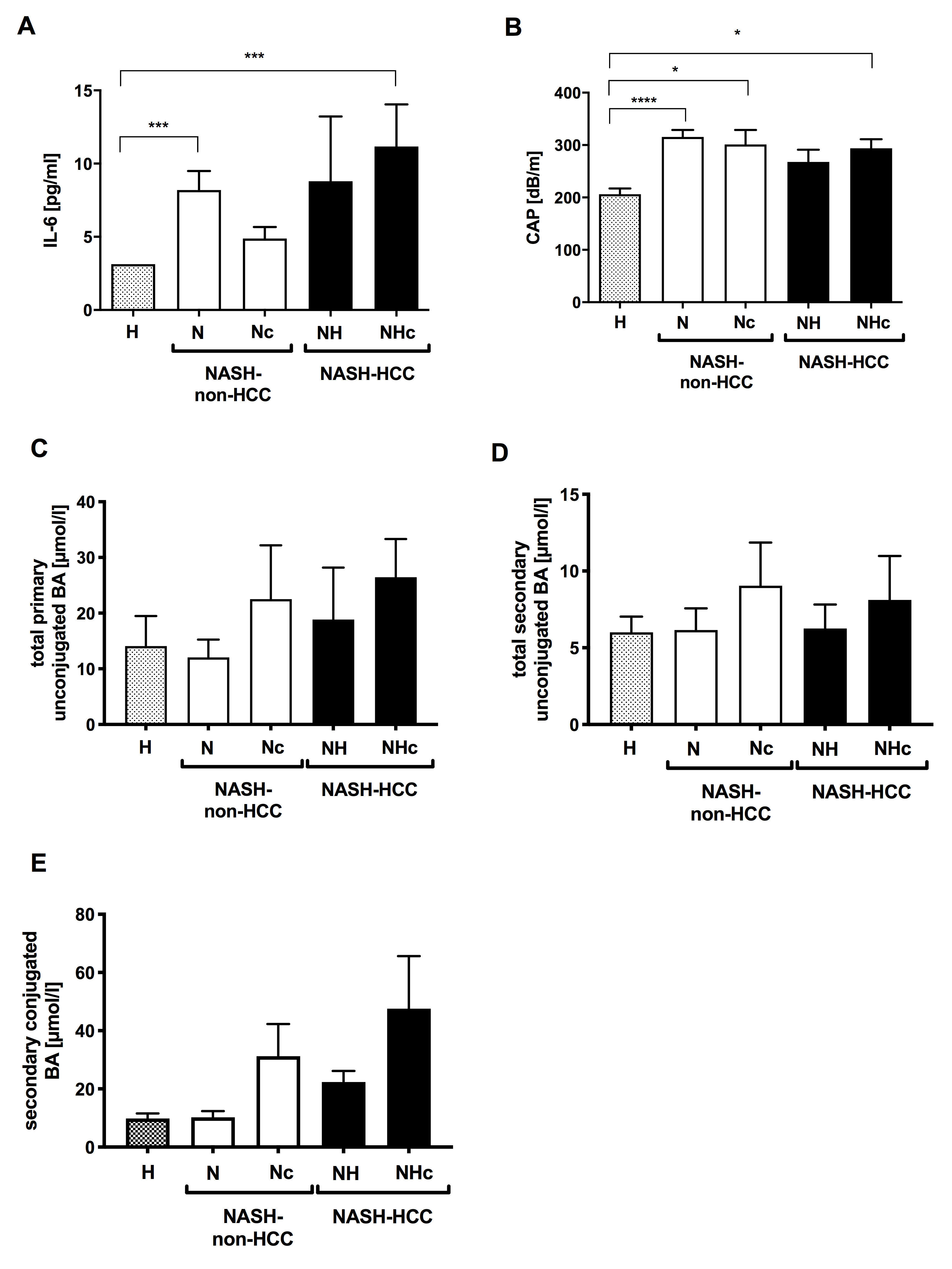

Supplement: SUPPLEMENTARY MATERIAL [file ct9-11-e00131-s003.tif]

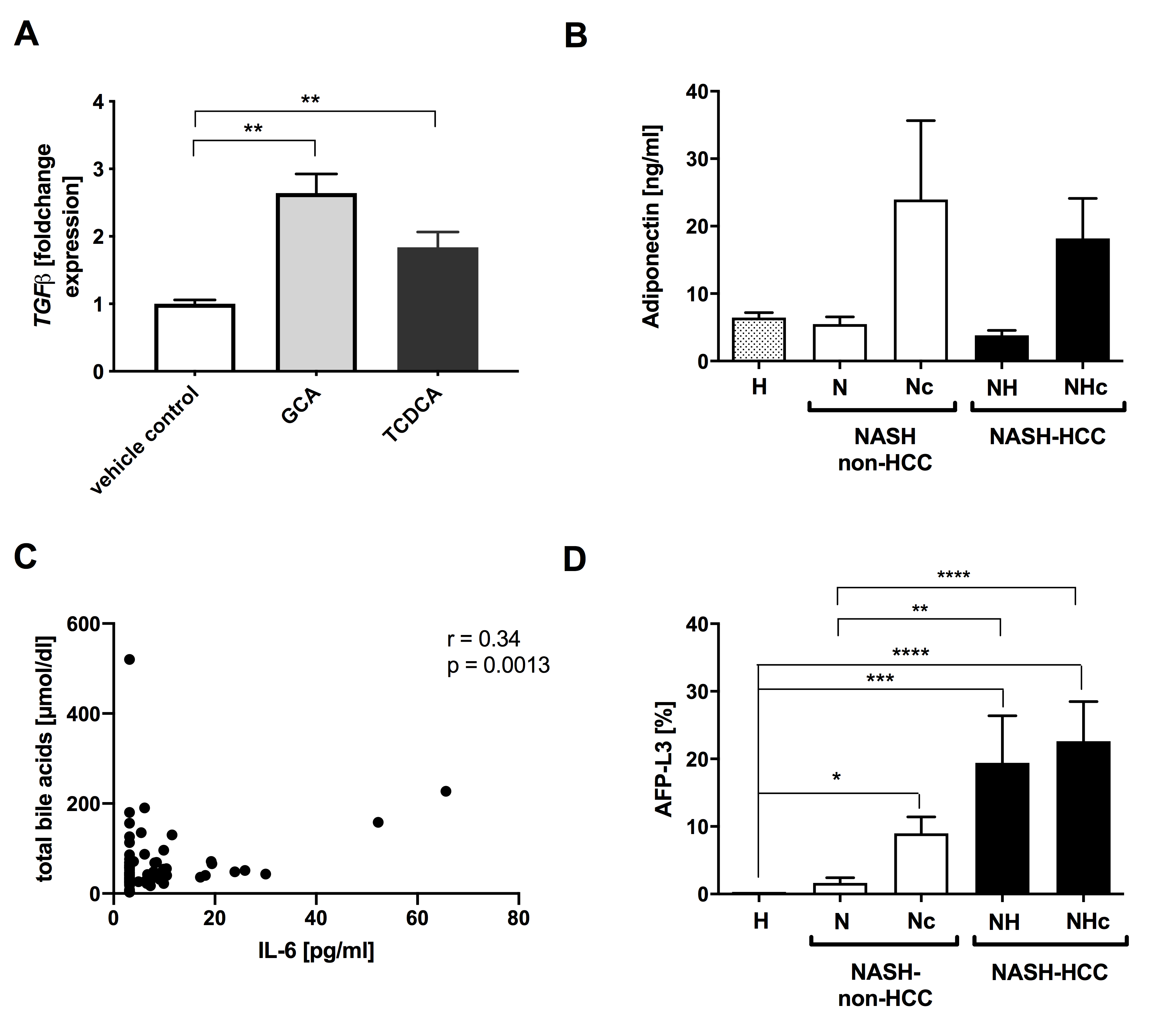

Supplement: SUPPLEMENTARY MATERIAL [file ct9-11-e00131-s004.tif]

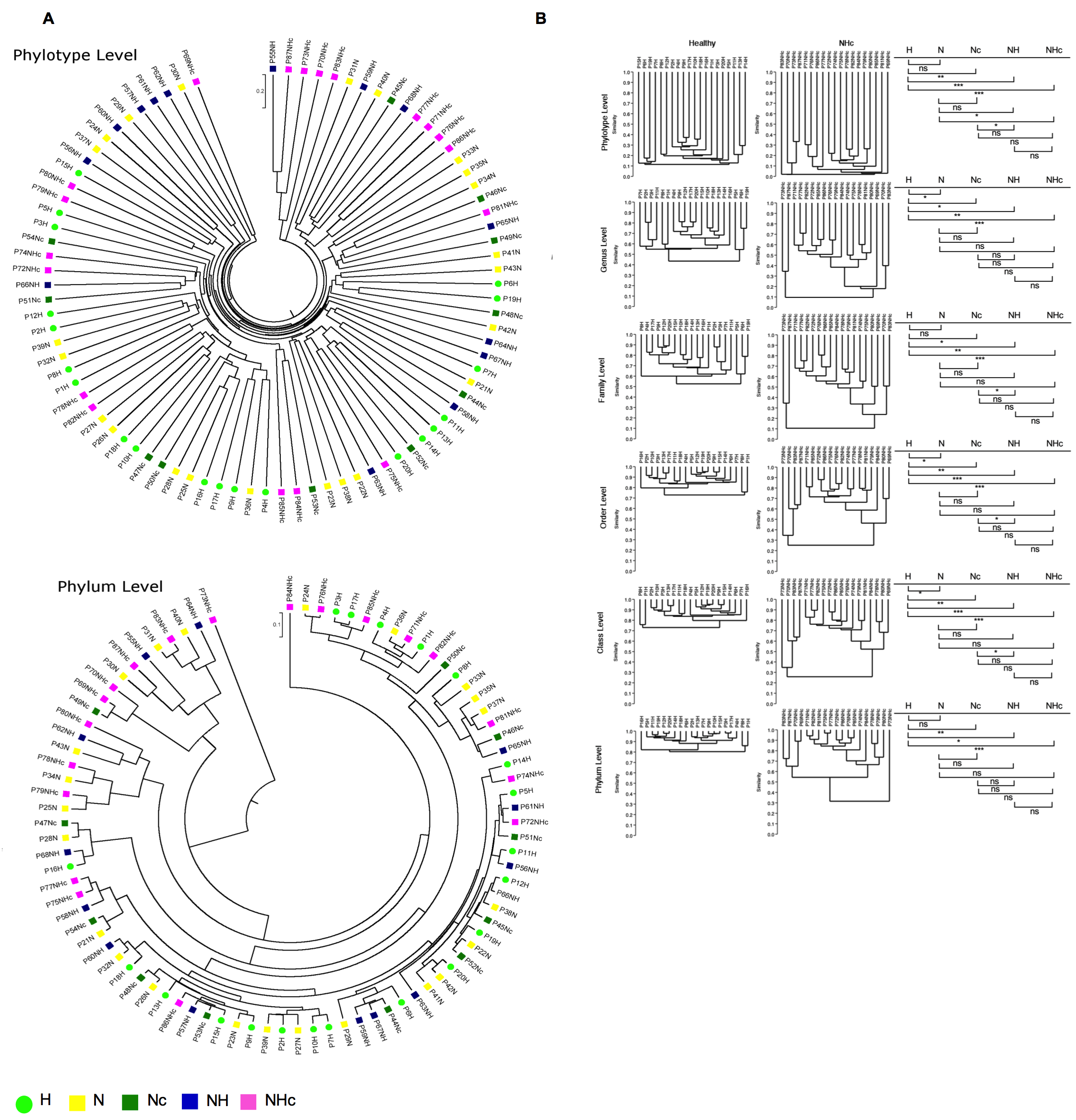

Supplement: SUPPLEMENTARY MATERIAL [file ct9-11-e00131-s005.tif]
